# Supplementary material for: Assessment of the Biological Impact of SARS-CoV-2 Genetic Variation Using an Authentic Virus Neutralisation Assay with Convalescent Plasma, Vaccinee Sera, and Standard Reagents
Source: Viruses. 2023 Feb 25;15(3):633. doi: 10.3390/v15030633 (PMC10056478; doi:10.3390/v15030633)
Supplement: Supplementary file 1 [file viruses-15-00633-s001.zip › Coombes et al - Supplementary table and figure - final.pdf]

Table S1 – Summary of neutralisation titres in a SARS-CoV-2 ancestral pseudotyped lentivirus virus (PSV) and focus-reduction (FRNT) neutralisation assay for a panel of 11 pre-Alpha convalescent plasma.

Sample ID	Harvest date	PSV (IC_{90})	FRNT (ND_{50})
NIBSC 7	May 2020	2321	13554
NIBSC 24	May 2020	1891	2684
NIBSC 31	June 2020	351	2033
NIBSC 32	May 2020	1137	4181
NIBSC 47	June 2020	101	773
NIBSC 61	May 2020	352	2238
NIBSC 78	June 2020	365	5309
NIBSC 80	June 2020	116	3122
NIBSC 82	June 2020	345	2889
NIBSC 83	June 2020	77	2832
NIBSC 86	June 2020	88	1920

Table S2 – Summary of geometric mean neutralisation titres from a focus-reduction assay (FRNT) for a panel of 11 pre-Alpha convalescent plasma against SARS-CoV-2 variants and their fold-changes relative to ancestral virus. Results are combined (geometric mean) from two independent laboratories and ordered by date of emergence.

Variant	Geometric mean of titres (ND_{50})	Fold-change relative to ancestral virus	95% confidence intervals
Ancestral	2,940	NA	1830 – 4720
Alpha	563	5.2*	359 – 883
Beta	106	27.6*	69.5 – 162
Gamma	2,690	1.1	1780 – 4070
Gamma - FC	750	3.9*	565 – 996
Alpha + E484K	522	5.6*	360 – 756
Zeta - FC	73.6	39.9*	43.1 – 126
Zeta - BEI	255	11.5*	146 – 444
Kappa	208	14.1*	120 – 363
Delta	318	9.2*	149 – 679
Delta - AY.1	310	9.5*	166 – 580
Delta - AY.4.2	338	8.7*	156 – 735
Lambda	350	8.4*	172 – 711
Mu	90.2	32.5*	60.5 – 134
Omicron - BA.1	24.5	120*	21.4 – 28.0
Omicron - BA.2	47.3	62.0*	29.9 – 74.8
Omicron - BA.1.1	23.8	123*	20.1 – 28.2
Recombinant - XF	24.8	118*	19.8 – 31.1
Omicron - BA.2.12.1	44.0	66.7*	27 – 71.9
Omicron - BA.4	31.5	93.2*	22.2 – 44.6
Omicron - BA.5.2.1	31.8	92.2*	23.1 – 43.9

Significant difference ($p<0.05$), as determined by a two-way ANOVA with Tukey's HSD post hoc test are indicated by *.

Table S3 – Summary of geometric mean neutralisation titres from a focus-reduction assay (FRNT) for a panel of 10 triple vaccinated human sera against SARS-CoV-2 variants and their fold-changes relative to ancestral virus. Results are ordered by date of variant emergence.

Variant	Geometric mean of titres (ND_{50})	Fold-change relative to ancestral virus	95% confidence intervals
Ancestral	5,230	NA	3444 – 7930
Omicron - BA.1	182	28.7*	97.4 – 340
Omicron - BA.2	199	26.2*	142 – 280
Omicron - BA.1.1	144	36.4*	93.4 – 221
Recombinant - XE	162	32.3*	114 – 229
Recombinant - XF	151	34.5*	89.5 – 256
Omicron - BA.2.12.1	166	31.4*	115 – 241
Omicron - BA.4	104	50.2*	71.1 – 152
Omicron - BA.5.2.1	65.4	79.9*	43.2 – 98.8
Omicron - BA.2.75.3	168	31.0*	96.3 – 295

Significant difference ($p<0.05$), as determined by a two-way ANOVA with Tukey's HSD post hoc test are indicated by *.

Table S4 – Summary of geometric mean neutralisation titres from a focus-reduction assay (FRNT) for a panel of 9 triple vaccinated human sera (excluding the participant with a breakthrough infection) against SARS-CoV-2 variants and their fold-changes relative to ancestral virus. Results are ordered by date of variant emergence.

Variant	Geometric mean of titres (ND_{50})	Fold-change relative to ancestral virus	95% confidence intervals
Ancestral	4580	NA	3280 – 6400
Omicron - BA.1	157	29.2*	86.0 – 286
Omicron - BA.2	182	25.2*	134 – 248
Omicron - BA.1.1	135	34.0*	84.9 – 214
Recombinant - XE	147	31.2*	108 – 199
Recombinant - XF	131	34.9*	81.8 – 211
Omicron - BA.2.12.1	158	28.9*	106 – 237
Omicron - BA.4	95.9	47.8*	65.6 – 140
Omicron - BA.5.2.1	57.2	80.1*	41.4 – 79.0
Omicron - BA.2.75.3	140	32.8*	92.1 – 212

Significant difference ($p<0.05$), as determined by a two-way ANOVA with Tukey's HSD post hoc test are indicated by *.

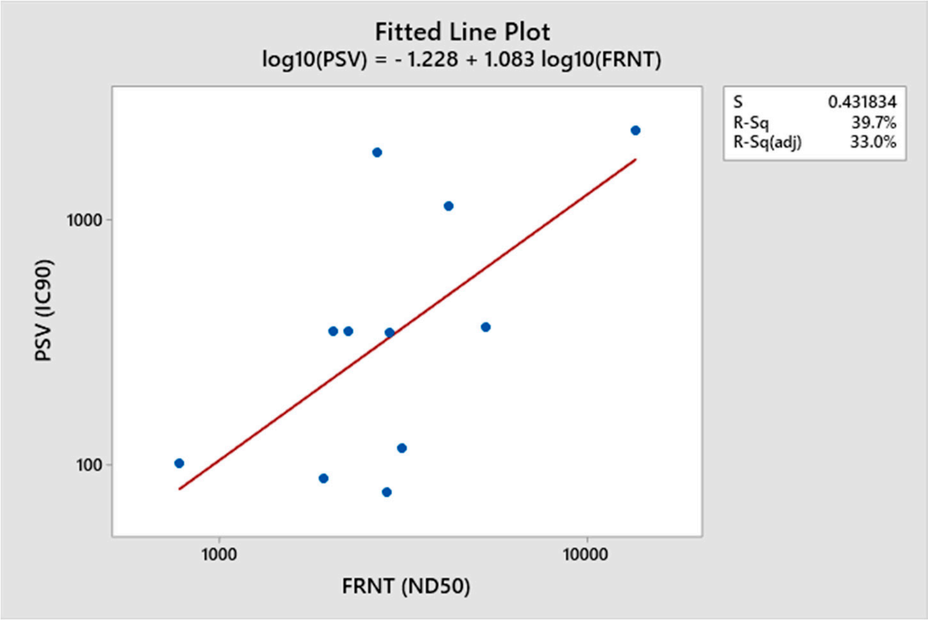

Figure S1. Regression analysis of neutralisation titres from an ancestral SARS-CoV-2 pseudotyped assay and focus-reduction neutralisation test (FRNT) for a panel of 11 pre-Alpha convalescent plasma

Regression analysis of 11 pre-Alpha convalescent plasma used for variant escape assessment showing the correlation (Pearson's r=0.63; p=0.038) between the two assays.

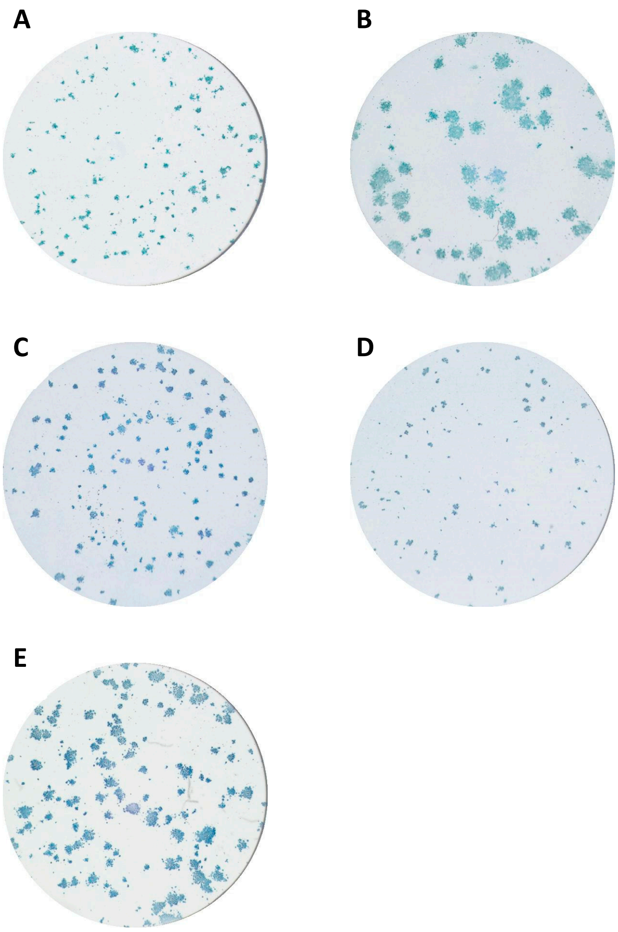

Figure S2. Foci morphology differences between SARS-CoV-2 variants used in this study

Vero-E6 cells infected with SARS-CoV-2 ancestral (A), Beta (B), Gamma (C) Omicron BA.1 (D) and Omicron BA.5 (E) variants as part of a focus forming assay with authentic virus. Immunostaining was performed with an anti-RBD antibody for authentic and Beta or anti-NC antibody for Omicron. Post-infection fixation time was optimised to 24hr (Ancestral), 20hr (Beta and Gamma), 26hr (Omicron BA.1) or 22h (Omicron BA.5).
